# Working Women in Large Towns

**Published:** 1890-12-20

**Authors:** 


### Working Women in Large Towns.

XVIII.—AIDS FOR WORKING WOMEN.—(*Continued.*)

*Sick Benefit Clubs.*—Comparatively little has as yet been done to provide for working women in times of sickness. The Trades Unions make provision for their members in this respect—by means, of course, of regular contributions in times of health; and a Women's Friendly Society has been started within the last few years at Long Melford, in Suffolk. It has spread to several other towns, and a branch (for what we may term professional working women) has lately been established in London, under the presidency of the well-known editor of *Work and Leisure.*

The National Deposit Friendly Society, again, has a special branch for women. It serves the purpose of a bank as well as a club, and is an excellent society, being well spoken of by that great authority on clubs, Canon Blackley; but we fear the number of female members is but small.

*Training Homes.*—We have spoken already of the great need of our working women—better technical training. To some, but to an extremely limited, extent, this need is supplied by the training-homes for domestic service, laundry-work, etc., in connection with the Metropolitan Association for Befriending Young Servants, and other charitable agencies, and by the various classes held at Homes and Clubs for working girls. But these are as a drop or two in an empty cistern; and we can but repeat that until more is done to secure practical education in our Board Schools, and to establish more schools for technical training, multitudes of our women must needs remain unskilled workers.

*Homes for Working Girls.*—It is difficult to speak too warmly of these. For girls who have bad homes, or no homes at all, it is everything to find the doors of such a bright, comfortable, happy place as "Norfolk House," or "The Welcome," open to her. She can there secure for herself comfortable board and lodging, useful classes, amusements, opportunities for putting by her savings and providing for medical attendance, cheerful companions, and sympathising friends in time of need. One has only to read the report of the committee on Mr. John Shrimpton's Homes (of which Norfolk House is one), to be convinced that they are doing very valuable work.

They prove of great service, again, as do other Homes in connection with the Young Women's Christian Association, the Girls' Friendly Society, etc., in providing a safe and comfortable refuge for servants who are out of place, and who, failing such a refuge, might easily fall into danger from sheer ignorance and helplessness. The Registry Offices connected with these Homes, and with the Metropolitan Association for Befriending Young Servants, also deserve special mention. Those who know how reckless, fraudulent, and, indeed, nothing short of infamous, some apparently respectable registries are, must needs bless those ladies who will be at the pains of finding out suitable places for young, ignorant, and perhaps giddy girls.

The Travellers' Aid Society, again, does excellent work, undertaking to meet young women at railway stations, and caring for their interests generally, until they have met with suitable homes.

*Evening Clubs.*—These clubs provide working girls with pleasant rooms in which they may spend the evenings with their "mates," attending useful classes, or amusing themselves according to their several tastes. Such a club is as excellent in its way as are the Homes mentioned above, and has a wonderful influence for good over the rough, untamed girls who flock into it. If well managed, it may truly be said to supply their every need, bodily, mental, and spiritual, even providing for pleasant intercourse between the members and their sweethearts. We have already mentioned that these clubs, by giving the girls a kind of second home, tend to prevent early marriage.

*The People's Palace.*—It is pleasant to know that this is largely patronised by City and East End work-girls, who are delighted to have the chance, not only of refreshing and benefiting both mind and body in class rooms and gymnasiums, but of acquiring technical knowledge in various industries. We trust that this boon of the East End will continue to receive generous support, and that the carrying out of plans for providing "Palaces of Delight" for other parts of London will not be hindered by lack of funds.

Space will not permit of our making this list of Aids for Working Women anything like an exhaustive one, though we think it is fairly representative of the lines proceeded upon by their sensible friends and well-wishers. We must, however, remind our readers that Parliament has already done something for women by passing the Factory Acts, thus protecting large classes of workers from excessive hours of toil. Philanthropists and reformers are apt to call out, directly they see any distress, that Parliament should step in and "do something," quite losing sight of the fact that it has already interfered in more than one direction, and that what is really wanted is, not new laws, but the better enforcement of laws already existing. With regard to two of the most important needs of our working women—proper hours of toil, and decently healthy workrooms—there are laws already existing which, if properly carried out, would do all, or nearly all, that is required. Let us agitate therefore, not for new laws, but for the better enforcement of the old ones. If the number of inspectors is not large enough (and everyone knows that is ludicrously insufficient), let us urge that it should be increased; or if Parliament must be appealed to, let it be asked for provisions which shall make the present laws easier of enforcement (such, for example, as the compulsory registration of workrooms, suggested by Mr. Booth) before we press for changes which may but aggravate, rather than relieve, our present distress.

